# Analytical and Numerical Modelling of Creep Deformation of Viscoelastic Thick-Walled Cylinder with Fractional Maxwell Model

**DOI:** 10.3390/ma14174849

**Published:** 2021-08-26

**Authors:** Xiang Ding, Na Chen, Yan Zhang, Fan Zhang

**Affiliations:** 1 School of Civil Engineering Architecture and Environment, Hubei University of Technology, Wuhan 430068, China; cn_research@hbut.edu.cn (N.C.); fanzhang@hbut.edu.cn (F.Z.); 2 Sino-French Joint Research Center of Rock, Soil Mechanics & Concrete Materials, Hubei University of Technology, Wuhan 430068, China; 3 School of Petroleum Engineering, Wuhan Campus, Yangtze University, Wuhan 430100, China; yanzhang1018@163.com

**Keywords:** thick-walled cylinder, viscoelastic, fractional Maxwell model, corresponding principle, numerical simulation

## Abstract

The deformation of a thick-walled cylinder under pressure is a classic elastic mechanics problem with various engineering applications. In this study, the displacement of a viscoelastic thick-walled cylinder under internal pressure is investigated via analytical as well as numerical modelling. The fractional Maxwell model is initially introduced to describe the creep deformation of high-strength Q460 steel. Subsequently, an analytical solution to the creep deformation of the thick-walled cylinder under both internal and external pressures is deduced with the corresponding principle. The analytical solution is examined with a numerical simulation that incorporates the fractional Maxwell model by a user-defined subroutine. The numerical simulation agrees well with the analytical solution. The limitations of the current study are also discussed.

## 1. Introduction

Thick-walled cylinders are widely used in the petrochemical industry, in natural gas, in high-pressure hydraulic systems and in other structures, such as high-pressure oil pipes, petrochemical pressure vessels, heat exchange tubes, storage vessels, nuclear reactor pressure vessels, cannon barrel, steam pipelines and functionally graded materials [1,2]. These internally pressured, thick-walled cylindrical vessels usually operate under high-temperature and high-pressure steam conditions; thus, creep deformation is considered one of the main failure mechanisms of these structures.

The creep deformation of thick-walled cylinders has been intensively investigated. Shinozuka [3] studied the stresses in an incompressible viscoelastic–plastic thick-walled cylinder. Attia et al. [4] studied the residual stresses produced in cast iron cylinders by the creep relaxation of thermal stresses. Schwiebert [5] proposed equations of stress rates in thick-walled cylinders subjected to transient thermal and mechanical loading. Pai [6] used a piecewise linear model to obtain solutions to the steady-state creep of a thick-walled orthotropic cylinder subjected to internal pressure. Sim and Penny [7] used reference stress techniques to investigate the plane strain creep behavior of thick-walled cylinders. Bhatnagar et al. [8,9,10,11] investigated the creep behavior of a thick-walled cylinder under various conditions. Simonian [12] calculated the thermal stresses in thick-walled cylinders, taking into account non-linear creep. Brust and Leis [13] proposed a model for predicting primary creep damage in axial cracked cylinders. Combescure [14] proposed a simplified method for the prediction of creep buckling in cylinders under external pressure. Orlando and Gonçalves [15] derived the lower bound to the creep rupture time of internally pressurized thick cylinders. Most of the above research studies considered thick-walled cylindrical vessels or cylinders with classical creep models. In contrast, few publications can be found which deal with creep in thick-walled cylindrical vessels or cylinders with more powerful fractional modelling techniques.

Thus, in this study, a fractional rheological model is first introduced to describe the creep deformation of Q460 steel under different uniaxial tensile stresses. With this fractional creep model, the creep deformation of a thick-walled cylinder subjected to internal pressure is analyzed through both analytical and numerical modelling.

## 2. Materials

Until the first application of fractional calculus in the solution of an integral equation by Abel, it has been a long time since the discussion of fractional calculus between L’Hôpital and Leibniz in 1695 [16,17]. Nowadays, fractional calculus is applied in many science and engineering territories, such as fluid flow, rheology, diffusion, electrical networks, electromagnetic theory and probability. The application of fractional calculus in rheology has been verified to be successful and various fractional rheological models have been proposed to describe the time-dependent phenomena [18]. Among these fractional models, due to its simplicity, the fractional Maxwell model has often been adopted to depict the rheological dynamics for polymers [19], foods [20], fluids [21] and geomaterials [22,23,24]. As fractional modelling is still unfamiliar to non-mathematicians, the constitutive equation for the fractional Maxwell model is presented briefly in the following section.

### 2.1. Constitutive Equations

**Figure 1:**
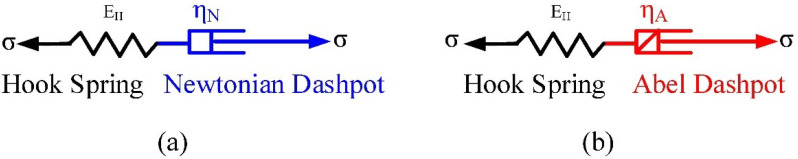
Illustration of the (**a**) integer Maxwell model; (**b**) fractional Maxwell model.

The traditional integer Maxwell model is composed of a spring element and a Newtonian dashpot in series connection, as illustrated in Figure 1a. The total strain of the Maxwell model includes the instantaneous elastic strain of the Hooke spring and the viscous strain of the Newtonian dashpot. Different from the traditional integer Maxwell model, the fractional Maxwell model [25,26,27,28,29], as shown in Figure 1b, replaces the Newtonian dashpot in the integer model with the Abel dashpot.

According to the constitutive relationship of the Abel dashpot and Hooke spring, and their series connection in the fractional Maxwell model, the constitutive equations of this fractional model can be expressed as follows: the total strain of the FMM is composed of the elastic strain represented by the Hooke spring and the viscous strain represented by the Abel dashpot. With the elastic constitutive equation of the Hooke spring and the fractional constitutive equation of the Abel dashpot, the constitutive relation of the FMM is expressed as

(1) $$\left\{\begin{array}{c} \varepsilon =\varepsilon_{e}+\varepsilon_{v}\;\;\\ \sigma =\sigma_{e}=\sigma_{v}\\ \sigma_{e}=E\varepsilon_{e}\;\;\\ \sigma_{v}=\eta^{\alpha }\frac{d^{\alpha }\varepsilon_{v}}{dt^{\alpha }} \end{array}\right.$$

in which ε, $\varepsilon_{e}$ and $\varepsilon_{v}$ are the total strain, elastic strain and viscous strain, respectively; σ, $\sigma_{e}$ and $\sigma_{v}$ are the stresses corresponding to the total strain, elastic strain and viscous strain, respectively; α is the fractional order of the Abel dashpot; *E* is the elastic modulus of the spring; *t* is the time; $\eta^{\alpha }$ is the viscosity of the dashpot and the superscript α is utilized to fulfil the principle of dimensional homogeneity.

After simplification, Equation (1) can be rewritten as

(2) $$\frac{d^{\alpha }\sigma }{dt^{\alpha }}+\frac{E}{\eta^{\alpha }}\sigma =E\frac{d^{\alpha }\varepsilon }{dt^{\alpha }}$$

Equation (2) can be rewritten with the Laplace transform technique as

(3) $$s^{\alpha }\sigma \left(s\right)+\frac{E}{\eta^{\alpha }}\sigma \left(s\right)=Es^{\alpha }\varepsilon \left(s\right)$$

where *s*, $\sigma \left(s\right)$ and $\varepsilon \left(s\right)$ are the complex variable, stress and strain in the Laplace domain, respectively. The creep modulus $J\left(s\right)$ in the Laplace domain is expressed with stress and strain as follows [25]:

(4) $$J\left(s\right)=\frac{\varepsilon \left(s\right)}{s\sigma \left(s\right)}$$

Thus, Equations (3) and (4) give

(5) $$J\left(s\right)=\frac{\varepsilon \left(s\right)}{s\sigma \left(s\right)}=\frac{s^{\alpha }\sigma \left(s\right)+\frac{E}{\eta^{\alpha }}\sigma \left(s\right)}{Es^{\alpha }\cdot s\sigma \left(s\right)}=\frac{1}{Es}+\frac{1}{\eta^{\alpha }}\frac{1}{s^{\alpha +1}}$$

Applying the inverse Laplace transform to Equation (5) yields

(6) $$J\left(t\right)=\frac{1}{E}+\frac{1}{\eta^{\alpha }}\frac{t^{\alpha }}{\Gamma \left(1+\alpha \right)}$$

where $J\left(t\right)$ is the creep modulus of FMM in the time domain.

If the fractional order in Equation (6) is 1, then Equation (6) reduces to

(7) $$J\left(t\right)=\frac{1}{E}+\frac{t}{\eta }$$

The expression of $J\left(t\right)$ in Equation (7) is the creep modulus of the IMM. In other words, the IMM is a special case of the FMM when α equals 1.

### 2.2. Fitting with Fractional Maxwell Model

To verify the practicability of the fractional Maxwell model introduced above, a group of strain–time curves obtained from creep tests of high-strength Q460 steel at various stress levels [30] are digitalized and fitted with the fractional Maxwell model. The creep strain is obtained by multiplying the creep modulus by the constant stress, as shown in Equation (8).

(8) $$\varepsilon \left(t\right)=J\left(t\right)\sigma_{0}$$

**Figure 2:**
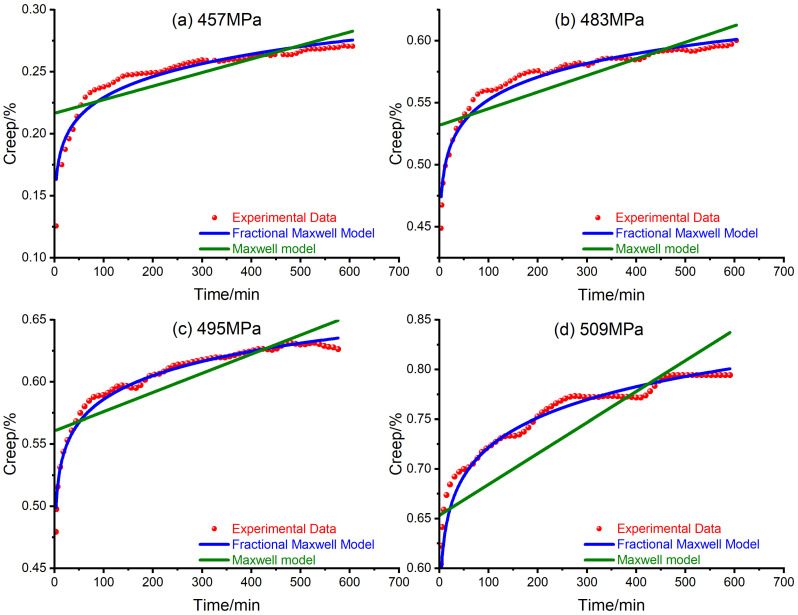
Fitting creep curves of Q460 steel with the Maxwell model and the fractional Maxwell model under various stress levels: (**a**) 457 MPa; (**b**) 483 MPa; (**c**) 495 MPa; (**d**) 509 MPa.

To show the superiority of the fractional Maxwell model, the traditional integer Maxwell model is also utilized to fit the creep curves of Q460 steel, as shown in Figure 2. The fitting parameters are also listed below in Table 1.

From Figure 2, the fractional Maxwell model, illustrated by the blue curve, is found to agree well with the experimental data, illustrated by the red dots, while the traditional Maxwell model, illustrated by the green lines, does not show close consistency with the experimental data. It can also be noted that both the traditional and fractional Maxwell model predict an ever-increasing creep strain; however, there is also a slight difference in that the traditional Maxwell model indicates a constant creep strain rate while the fractional Maxwell model indicates a decreasing creep strain rate. Compared with the traditional Maxwell model, the fractional Maxwell model achieves a better fitting result with one more parameter α; therefore, the fractional Maxwell model seems an appropriate candidate to simulate the viscoelastic deformation of steel.

## 3. Methods

**Figure 3:**
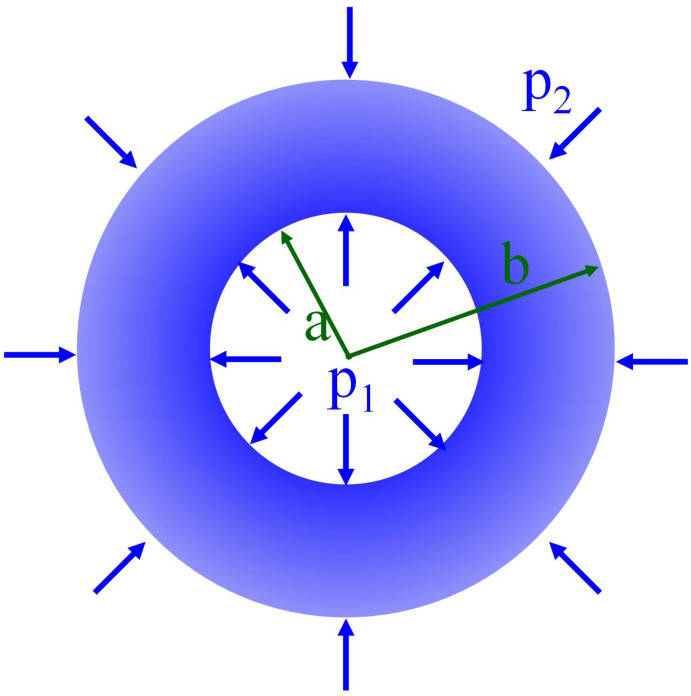
Illustration of a pressured thick-walled cylinder.

Figure 3 illustrates a pressured thick-walled cylinder under both internal and external pressures. Under the plane stress condition, the elastic solution of the radial displacement of the cylinder can be expressed as [31]

(9) $$u\left(r\right)=\frac{1}{2G}\left[\frac{\left(\kappa -1\right)\left(b^{2}p_{2}-a^{2}p_{1}\right)r}{2\left(b^{2}-a^{2}\right)}-\frac{a^{2}b^{2}\left(p_{1}-p_{2}\right)}{\left(b^{2}-a^{2}\right)r}\right]$$

where *a* and *b* are the inner and outer radius of the cylinder, respectively; $p_{1}$ and $p_{2}$ are the internal and external pressures, respectively; κ is a parameter depending on plane stress or plane strain conditions; *G* is the shear modulus and *r* is the distance from any point within the cylinder to the center of the cylinder. When it comes to the plane strain condition, the radial displacement can be obtained by replacing κ with 3−4v in Equation (9) as

(10) $$u\left(r\right)=\frac{1}{G}\left[\frac{\left(b^{2}p_{2}-a^{2}p_{1}\right)r}{2\left(b^{2}-a^{2}\right)}\left(1-2\nu \right)-\frac{a^{2}b^{2}\left(p_{1}-p_{2}\right)}{\left(b^{2}-a^{2}\right)r}\right]$$

Hooke’s law is decomposed into isotropic and deviatoric parts as

(11) $$\left\{\begin{array}{c} \sigma_{m}=3K\varepsilon_{m}\\ s=2Ge \end{array}\right.$$

where $\sigma_{m}$, $\epsilon_{m}$ are mean normal stress and strain, respectively; s, e are deviatoric stress/strain tensor, respectively; *K*, *G* are volume and shear modulus, respectively. The viscoelastic generalization of Hooke’s law can be expressed as

(12) $$\left\{\begin{array}{c} f_{1}\left(D\right)\sigma_{m}=3g_{1}\left(D\right)\varepsilon_{m}\\ f\left(D\right)s=2g\left(D\right)e \end{array}\right.$$

where the operator *D* is interpreted as representing partial differentiation with respect to time; *f*, *g*, $f_{1}$, $g_{1}$ are polynomials in *D*. The Laplace transforms of the viscoelastic generalization of Hooke’s law are

(13) $$\left\{\begin{array}{c} f_{1}\left(s\right){\tilde{\sigma }}_{m}=3g_{1}\left(s\right){\tilde{\varepsilon }}_{m}\\ f\left(s\right)\tilde{s}=2g\left(s\right)\tilde{e} \end{array}\right.$$

Suppose that the solution to a certain problem in elasticity is available; then, the associated viscoelastic solution in which the same stresses are applied at t=0 to an initially undisturbed body can be obtained by replacing *K* with $g_{1}\left(s\right)/f_{1}\left(s\right)$ and *G* with g(s)/f(s). The actual stresses and strains in the time domain can then be acquired through inverse Laplace transforms.

Now, suppose that the material is elastic in hydrostatic compression and follows the fractional Maxwell model in shear. Equation (3) can be rearranged as

(14) $$\left(s^{\alpha }+\frac{E}{\eta^{\alpha }}\right)\tilde{\sigma }=Es^{\alpha }\tilde{\varepsilon }$$

Comparing Equations (13) and (14), the shear modulus is expressed as

(15) $$G=\frac{g\left(s\right)}{f\left(s\right)}=\frac{Es^{\alpha }}{s^{\alpha }+\frac{E}{\eta^{\alpha }}}$$

Replacing *G* with Equation (15), the displacement in the Laplacian domain is expressed as

(16) $$u\left(s\right)=\frac{s^{\alpha }+\frac{E}{\eta^{\alpha }}}{Es^{\alpha }}\left[\frac{\left(b^{2}{\tilde{p}}_{2}-a^{2}{\tilde{p}}_{1}\right)r}{2\left(b^{2}-a^{2}\right)}\left(1-2\nu \right)-\frac{a^{2}b^{2}\left({\tilde{p}}_{1}-{\tilde{p}}_{2}\right)}{\left(b^{2}-a^{2}\right)r}\right]$$

where ${\tilde{p}}_{1}$ and ${\tilde{p}}_{2}$ are Laplace transforms of constant stress $p_{1}$ and $p_{2}$. Thus, ${\tilde{p}}_{1}$ and ${\tilde{p}}_{2}$ are expressed as

(17) $$\left\{\begin{array}{c} {\tilde{p}}_{1}=\frac{p_{1}}{s}\\ {\tilde{p}}_{2}=\frac{p_{2}}{s} \end{array}\right.$$

With Equation (17), Equation (16) can be rewritten as

(18) $$u\left(s\right)=\left(\frac{1}{Es}+\frac{s^{-1-\alpha }}{\eta^{\alpha }}\right)\left[\frac{\left(b^{2}p_{2}-a^{2}p_{1}\right)r}{2\left(b^{2}-a^{2}\right)}\left(1-2\nu \right)-\frac{a^{2}b^{2}\left(p_{1}-p_{2}\right)}{\left(b^{2}-a^{2}\right)r}\right]$$

Then, the radial displacement in the time domain can be obtained with the inverse Laplace transform of Equation (18) as

(19) $$\begin{array}{c} u\left(t\right)=L^{-1}\left[u\left(s\right),s,t\right]\\ \;\;\;=L^{-1}\left\{\left(\frac{1}{Es}+\frac{s^{-1-\alpha }}{\eta^{\alpha }}\right)\left[\frac{\left(b^{2}p_{2}-a^{2}p_{1}\right)r}{2\left(b^{2}-a^{2}\right)}\left(1-2\nu \right)-\frac{a^{2}b^{2}\left(p_{1}-p_{2}\right)}{\left(b^{2}-a^{2}\right)r}\right],s,t\right\}\\ \;\;\;=\left[\frac{\left(b^{2}p_{2}-a^{2}p_{1}\right)r}{2\left(b^{2}-a^{2}\right)}\left(1-2\nu \right)-\frac{a^{2}b^{2}\left(p_{1}-p_{2}\right)}{\left(b^{2}-a^{2}\right)r}\right]\left(\frac{1}{E}+\frac{t^{\alpha }}{\eta^{\alpha }\Gamma \left(1+\alpha \right)}\right) \end{array}$$

It can be seen from Equation (19) that the radial displacement is composed of two parts—the elastic part and the time-dependent part. When t→0, the radial displacement reduces to the elastic component as

(20) $$u\left|{}_{t=0}\right.=\frac{1}{E}\left[\frac{\left(b^{2}p_{2}-a^{2}p_{1}\right)r}{2\left(b^{2}-a^{2}\right)}\left(1-2\nu \right)-\frac{a^{2}b^{2}\left(p_{1}-p_{2}\right)}{\left(b^{2}-a^{2}\right)r}\right]$$

Equation (20) is almost the same as Equation (10), with only the slight difference that the shear modulus *G* in Equation (10) is replaced by the elastic modulus *E* of the fractional Maxwell model in Equation (20), which can be explained by the basic assumption that the time-dependent deformation relies only on the shear stress, as stated previously.

## 4. Results

### 4.1. Numerical Implementation of Fractional Maxwell Model

Though the history of fractional calculus is long, it is still somewhat new to non-mathematicians and ordinary engineers. Fortunately, as a general finite element platform, ABAQUS offers users great flexibility to build their own models with a secondary development function. More specifically, when ABAQUS fails to provide an appropriate model to deal with a certain time-dependent material, user subroutine CREEP can be used to define the rheological behavior of this material [32].

If user subroutine CREEP is used to define a material’s behavior, the subroutine aims to offer the “uniaxial” creep laws, which will be incorporated into a general time-dependent material formulation. It can be coupled with temperature and electricity analysis. It also should be noted that the meaning of variables defined in this subroutine depends on the specific model chosen. Taking the variables DECRA(1), for example, it may represent the equivalent uniaxial deviatoric/cohesion/compressive creep strain increment when the subroutine CREEP coupled with the metal/capped Drucker–Prager/gasket model. Details of the numerical implementation of the fractional Maxwell model can also be found in a previous study [23] and the manual of the ABAQUS subroutine [32].

To check the reliability of the built-in creep subroutine of the fractional Maxwell model in ABAQUS, numerical creep experiments of high-strength Q460 steel at various stress levels are conducted and compared with the laboratory experiments carried out by Wang [30] et al.

**Figure 4:**
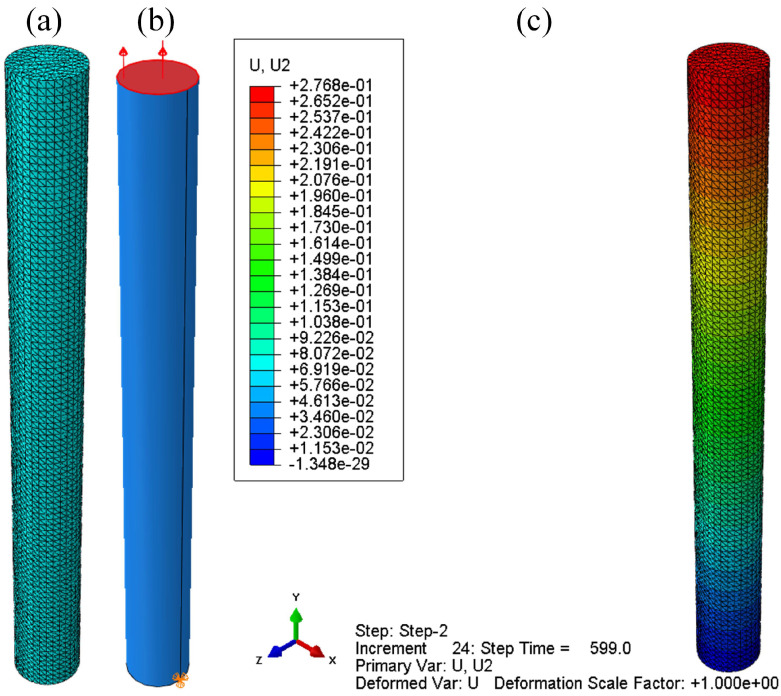
(**a**) Mesh scheme; (**b**) load and boundary conditions; (**c**) axial displacement at the end of load.

The geometrical model of the steel specimen is illustrated in Figure 4a. The steel cylinder with a diameter 10 mm is 100 mm in length. The steel bar is divided into 100 segments along the axial direction and 20 segments along the circumferential direction, as shown in Figure 4a. The C3D10 is adopted. There are 41,241 elements in total. The steel bar is fixed at the bottom and a tensile stress is applied at the top of the bar, as shown in Figure 4b. The whole simulation is divided into three steps, i.e., initial, elastic and viscoelastic, and the loading process is illustrated as shown in Table 2. In the elastic step, the axial tensile stress increases linearly to the design load and the load will hold constant during the viscoelastic step. Under the stress of 457 MPa, the axial displacement of the steel bar at the end of viscoelastic step is plotted as shown in Figure 4c.

**Figure 5:**
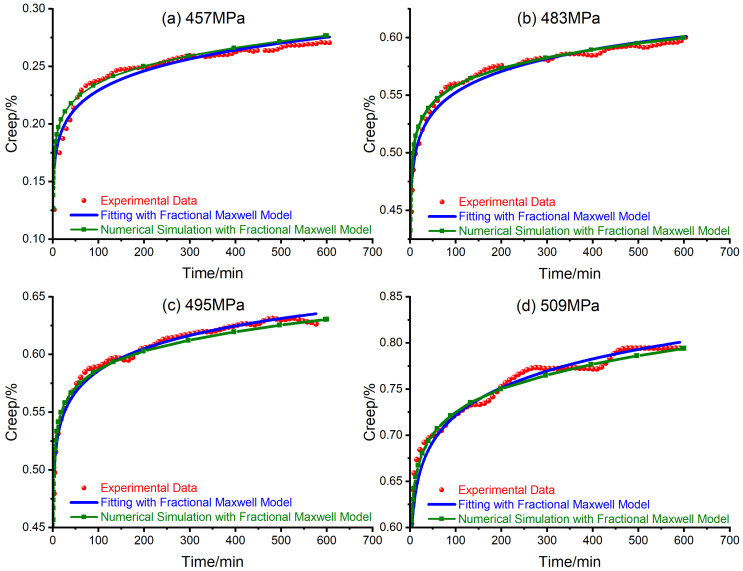
Creep strain of steel bar under different axial stress levels: (**a**) 457 MPa; (**b**) 483 MPa; (**c**) 495 MPa; (**d**) 509 MPa.

Repeating the simulation process mentioned above, with the mechanical parameters listed in Table 1, the creep of steel bars under different axial stresses is simulated as shown in Figure 5.

Form Figure 5, the numerical simulations reproduce the creep experiments of steel bars under different stress levels. Thus, the fractional Maxwell model seems to be a suitable candidate to simulate the first and second stage of the creep deformation of steel bars.

### 4.2. Numerical Simulation of Creep Deformation of Thick-Walled Cylinder under Internal Pressure

**Figure 6:**
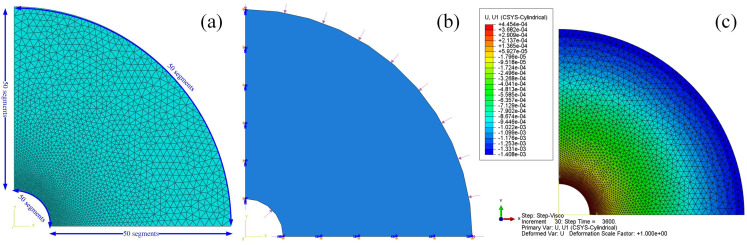
(**a**) Mesh scheme; (**b**) boundary condition; (**c**) radial displacement at the end of loading.

A plain strain model is established to simulate the creep deformation of a thick-walled cylinder under internal pressure. The inner and outer radii of the cylinder are 1 cm and 6 cm, respectively. The elastic modulus of cylinder is 10 GPa and the Poisson’s ratio is 0.3. The viscosity of the cylinder is $10^{13}\;{\mathrm{Pa}\cdot s}^{a}$ and the fractional order is 0.5. Each edge of the cylinder is divided into 50 segments. A bias mesh scheme is adopted on two straight edges and the bias ratio is 10, as shown in Figure 6a. The internal and external pressures with the magnitude 10 MPa and 5 MPa are applied on the inner and outer surface, respectively, and a symmetric condition is cast on the two straight edges, as shown in Figure 6b. The internal pressure is held for 3600 s. During the whole loading process, the radial displacement at the inner surface of the cylinder is recorded. The radial displacement at the end of loading is plotted as in Figure 6c. It should be noted that Figure 6c is plotted in a local cylindrical coordinate system.

**Figure 7:**
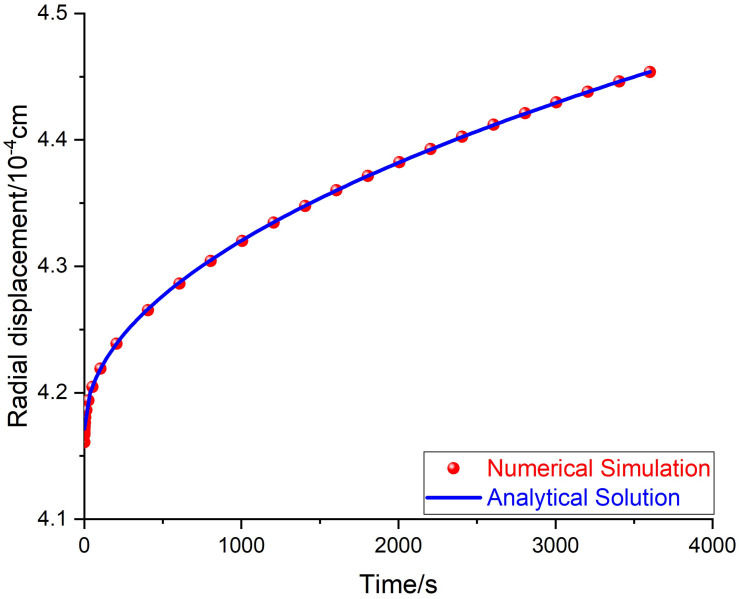
Numerical simulation and analytical solution of the radial displacement at the inner surface.

The theoretical radial displacement at the inner surface of the cylinder is plotted according to Equation (19) and radial displacement by the numerical simulation is also plotted with the data obtained at each step, and they are both plotted and compared in Figure 7. In Figure 7, the numerical simulation results almost coincide with the analytical solution predicted by Equation (19). The analytical solution of the displacement at the inner surface under constant pressure is validated by the numerical simulation.

## 5. Discussion

In line with the previous section, it should be noted that there are also limitations of the current study. Firstly, only the viscoelastic deformation is considered in this study and the plastic deformation is not taken into consideration. This is a requirement of the elastic–viscoelastic correspondence principle [33]. This is also a requirement of common practice for most containers and pipes that operate below the yield stress. Secondly, only the transient and the steady-state creep stages are considered both in the analytical solution and numerical simulation, and the acceleration creep stage is omitted for simplicity. Both the traditional integer Maxwell model and the fractional Maxwell model can only describe the transient and the steady-state creep stages; more complicated rheological models may be introduced to tackle the complexity involved with the acceleration creep stage. The numerical simulation with more complicated rheological models may not be overly difficult to implement; however, whether there would be analytical solutions generated by these models remains uncertain. The fractional Maxwell model seems to find a balance between more complicated models and practical needs, as the fractional Maxwell model captures the primary features of the steel creep and also enables an analytical solution of the displacement for the internally pressured, thick-walled cylinder.

## 6. Conclusions

The viscoelastic deformation of a thick-walled cylinder under internal pressure is investigated via analytical modelling and numerical simulation. Based on the study, the following conclusions are made:

1. Compared to the traditional integer Maxwell model, the fractional Maxwell model is an efficient nonlinear model to capture the essence of creep deformation of steels both in the transient and the steady-state stages.
2. In analogy with the analytical models based on traditional integer creep models, the analytical solutions based on fractional models can also be established with the help of the elastic–viscoelastic correspondence principle.
3. The implementation of the fractional Maxwell model with the help of the second development function enriches the material libraries of the general-purpose finite element software and enables the capability of finite element software to conduct more realistic numerical simulations.
4. The numerical simulation results validate the creep displacement of a thick-walled cylinder under constant internal pressure predicted by the analytical solution based on the fractional Maxwell model. Hence, the analytical solution of creep displacement of a thick-walled cylinder under constant internal pressure based on the fractional Maxwell model can be used with confidence to predict the time-dependent deformations.

## Figures and Tables

**Table 1:** Fitting parameters for creep models.

Stress/MPa	Creep Model				
	Fractional Maxwell Model			Maxwell Model	
	$E/10^{25}$ Pa	$\eta^{\alpha }/10^{11}\;\mathrm{Pa}\cdot h^{a}$	α	$E/10^{11}$ Pa	$\eta /10^{14}\;\mathrm{Pa}\cdot h$
457	7.01834	3.41866	0.10518	2.12553	4.03905
483	1.10758	1.11339	0.0469802	0.908136	3.60626
495	0.704099	1.07122	0.0461791	0.882804	3.2086
509	1.40625	0.952868	0.0585737	0.779106	1.63443

**Table 2:** Loading procedure for numerical simulation of creep experiment of steel bar.

Loading Step	Time/s	Amplitude	Initial Step/s	Minimum Step/s	Maximum Step/s
Initial_Loading	0	0	/	/	/
Elastic_Loading	1	1	10${}^{-2}$	10${}^{-7}$	0.1
Viscous_Loading	599	1	1	10${}^{-3}$	100

## Data Availability

Data sharing is not applicable to this article.
